# Public knowledge, attitude, and acceptance toward COVID-19 vaccines in Palestine: a cross-sectional study

**DOI:** 10.1186/s12889-022-12932-4

**Published:** 2022-03-17

**Authors:** Mohammed Al-kafarna, Sajeda Ghassan Matar, Hossam Waleed Almadhoon, Bashar Khaled Almaghary, Mohamed Sayed Zaazouee, Asmaa Ahmed Elrashedy, Dalia Sami Wafi, Sami D. Jabari, Omar H. Salloum, Eman Ahmed Ibrahim, Hala ZI Alagha, Elfatih A. Hasabo, IMedRA Collaboration Team, Thara Kh. Hussein AL-Ali, Haroun Neiroukh, Falasteen Jameel Almakhtoob, Sireen Sufian Marabeh, Malak Y. Badawi, Anwar Y. Jabari, Fida Hussien Al-Ali, Tamer Sami Asafrah, Yara Safwat Muhanna

**Affiliations:** 1 Faculty of Pharmacy, Al-Azhar University – Gaza, Gaza Strip, Palestine; 2 Faculty of Pharmacy, University of Jordan, Amman, Jordan; 3 Faculty of Dentistry, Al-Azhar University – Gaza, Gaza Strip, Palestine; 4 Faculty of Medicine, Al-Azhar University, Assiut, Egypt; 5 Faculty of Medicine, Kafr El-Shaikh University, Kafr El-Shaikh, Egypt; 6 Faculty of Medicine, Al-Azhar University – Gaza, Gaza Strip, Palestine; 7 Faculty of Medicine, Palestine Polytechnic University, Hebron, Palestine; 8 Assistant Professor, Department of Clinical Pharmacy, Faculty of Pharmacy, Al-Azhar University – Gaza, Gaza Strip, Palestine; 9 Faculty of Medicine, University of Khartoum, Khartoum, Sudan

**Keywords:** COVID-19, Pandemic, Vaccine, Knowledge, Attitude, Acceptance, Palestine

## Abstract

**Background:**

The consequences of the COVID-19 pandemic on physical and mental health in addition to the global economy are huge. Vaccination is a pivotal measure to decrease COVID-19 morbidity and mortality and to help bring the pandemic under control. Yet, success of the vaccination process depends on the population’s willingness to be vaccinated which may be determined by their level of knowledge about and trust in currently available COVID-19 vaccines. Therefore, this study aims to assess the knowledge, attitude, and acceptance of Palestinians towards COVID-19 vaccines.

**Methods:**

A national cross-sectional study was distributed in different Palestinian regions to assess the knowledge and attitude of Palestinians toward COVID-19 vaccines using an online questionnaire, it included three sections; sociodemographic characteristics, knowledge assessment questions, and attitude assessment questions.

**Results:**

A total of 6226 participants completed the questionnaire; among them, 41.36% believed that vaccines are safe, 69.02% agreed that vaccines are vital to protect from COVID-19; in addition, 55.1% approve administering the vaccine once available, and 37.86% do not believe their benefits outweigh the risks. The Source of information for 22.07% of participants in social media, while 11.92% rely on health care providers. Participants’ attitudes and knowledge were significantly affected by gender, governorate, age, education level, and marital status (*P* <0.001).

**Conclusion:**

The findings suggest that there is good knowledge and attitude toward the vaccination process against COVID-19 in Palestine, although low acceptance was detected. Awareness campaigns are required to spread reliable knowledge about COVID-19 vaccines.

## Introduction

The coronavirus disease of 2019 (COVID-19) is a disease first triggered at the end of 2019 in Wuhan (Hubei, China) caused by a virus called severe acute respiratory syndrome coronavirus 2 (SARS-CoV-2) [1–3]. The World Health Organization (WHO) declared on March 2020 a pandemic after deciphering the SARS-CoV-2 genomic sequence earlier in the same year [2, 3]. This pandemic has forced governments and policymakers to impose decisions of national lockdowns partially or completely and put billions of people in quarantine, which severely affected various aspects of life including psychological and economic impact [4, 5]. In addition to lockdowns, governments focused on other non-pharmaceutical interventions (NPIs) strategies to diminish the virus transmissibility including masks wearing, hand sterilizing, social distancing, schools, online education, and transfers restrictions [6].

By the end of May 2021, the WHO has registered more than 168 million confirmed cases of COVID-19 infection globally and more than 3.5 million deaths [7]. Concurrently, the scientific community has made unprecedented efforts to counteract the health crisis by conducting research projects on SARS-CoV-2 to understand its behavior, presenting the clinical image of COVID-19, and developing vaccine candidates [8–10]. The scientific community believes that vaccines are key strategy to stop the spread of the SARS-CoV-2 virus and end the pandemic. Accordingly, many pharmaceutical companies’ labs and research institutes have been working on the virus vectors including mRNA, DNA, subunit, and virus-like particles to develop a vaccine for COVID-19 [11, 12].

Over the past decades, vaccinations have been known to be an effective strategy in preventing the spread of certain viral infections [13]. Since vaccines are considered the most cost-effective and reliable public health interventions, which have been implemented over the past century and saved millions of lives each year [13–15]. Individual vaccination is considered an effective preventive measure as it provides direct immunity for the vaccinated individuals and prevents the spread of the disease among them. Also, vaccines have been proved to shrink the virus spread even among non-vaccinated individuals through herd immunity when sufficient immune proportions are available in the population [16].

Many countries have focused on accelerating the process of vaccine development, therefore, by the end of May 2021, more than 102 vaccines were in the clinical stage and more than 184 vaccines in the pre-clinical stage [17]. Despite the rapid progress of COVID-19 vaccine development, the public attitude toward vaccine acceptance is still challenging which affects the complete mitigation of the pandemic [18]. A study from Kuwait revealed that only 53.1% of participants were willing to receive COVID-19 vaccines once available and those who consider vaccines to have health related risks were less willing to accept vaccination [19]. For instance, 15.6% of people in Saudi Arabia would definitely not administer COVID-19 vaccine, while 26.8% of them would probably not accept the administration of the COVID-19 vaccine [20], while only 7% of people in Bangladesh would definitely not administer the COVID-19 vaccine and 24% of them probably won’t accept administering it [21]. Therefore, the readiness of people to accept the COVID-19 vaccine is vital to succeeding in the vaccination programs [22]. Hence, this national cross-sectional survey aims to measure the knowledge, attitude, and acceptance of the public population in Palestine toward COVID-19 vaccines.

## Methods

The study was conducted following the Checklist for Reporting Results of Internet E-Surveys (CHERRIES) guidelines [23].

### Study design and participants

This is a national, cross-sectional study to assess the knowledge, attitude, and acceptance toward COVID-19 vaccines in the different Palestinian regions using an online self-administered survey pre-piloted and pre-validated during the period 7 May to 6 June 2021. Palestinian who are currently living in Palestine and aged > 18 years were included, while participants age < 18 years old or who did not complete the questionnaire or refused to participate were excluded.

### Sampling

We used Epi info sample size calculator software to calculate the sample size, which is 384 by power 80% and confidence interval of 95%. To increase the validity of the study and decrease the standard error of a convenient sample approach, and to have more representative sample, a larger number of participants were recruited. Therefore, a total of 5979 participants were included in this study.

### Questionnaire development

The study questionnaire was developed and structured into three sections (sociodemographic, knowledge, and attitude) followed by validation and translation into Arabic. The first section consists of demographic characteristics including age, gender, residence, marital status, education, chronic diseases, previous COVID-19 infection, vaccine administration, source of vaccines information, and fears faced during the COVID-19 pandemic. The second section aims to assess the knowledge of people towards COVID-19 vaccines by 18 questions with a 3-Likert scale; Yes/ No /I don't know, we referenced knowledge questions according to El-Elimat et al. [24], while the third part aims to measure the attitudes of people towards COVID-19 vaccines through 9 questions with a 3-Likert scale, Agree/ Disagree /I don't know. Questions used to assess attitude are referenced according to Abdul et al. [25]. A pilot sample (*n*=30) was used to measure internal consistency and improve the clarity of the questionnaire items. The questionnaire showed strong internal consistency with Cronbach's alpha value (0.75).

### Data collection and handling

We recruited collaborators from different locations of Palestine to collect the data in a snowball fashion. The form was sent to social media platforms including Facebook, Instagram, Twitter, and WhatsApp. All collected data were stored confidentially on the author's laptop.

### Statistical analysis

IBM's Statistical Package for Social Sciences SPSS version 25 was used to analyze the data. Descriptive analysis (percentage and frequency) was used to describe demographic characteristics and the prevalence of answers for each question in the questionnaire. Furthermore, Mann-Whitney was used to compare the knowledge score and Gender, Governorate, Chronic disease, Previous coronavirus infection, and vaccine administration, while the Kruskal-Wallis test was used to compare Age group, Education level, and Marital status with knowledge score. *P*-values of less than 0.05 were considered statistically significant.

## Results

### Socio-demographic characteristics of participants

The study included 5979 Palestinians from various governorates in the Gaza Strip and the West Bank, 15.1% of participants were from Gaza and 84.9% were from the West Bank, 73.8% of participants were female, and most participants’ age was between 20 and 24 (47.7%). More than 75% of participants had a university education (92.9%), and single participants account for 78% of the study population. 96% of the participants did not have any chronic diseases. Approximately half (56.1%) of participants reported that they had previously been infected with COVID-19. Additionally, (85.3%) of the participants did not receive the vaccine (See Table 1).

**Table 1:** Socio-Demographic characteristics of participants included in the study

Variable	N (%)(*N*=5979)
Gender	
Male	1564 (26.2%)
Female	4415 (73.8%)
The Governorate	
Gaza strip	903 (15.1%)
West Bank	5076 (84.9%)
Age group	
18 – 19	2064 (34.5%)
20 – 24	2854 (47.7%)
25 – 29	348 (5.8%)
> 29	713 (11.9%)
Education level	
Non-educated	23 (0.4%)
Primary school	13 (0.2%)
Elementary school	38 (0.6%)
Secondary school	353 (5.9%)
University	5552 (92.9%)
Marital status	
Single	4662 (78%)
Married	1253 (21%)
Divorced	39 (0.7%)
Widower	25 (0.4%)
Chronic disease	
Yes	237 (4%)
No	5742 (96%)
Previously infected with coronavirus	
Yes	3357 (56.1%)
No	2622 (43.9%)
Administered a COVID-19 vaccine	
Yes	879 (14.7%)
No	5100 (85.3%)

**Fig. 1:**
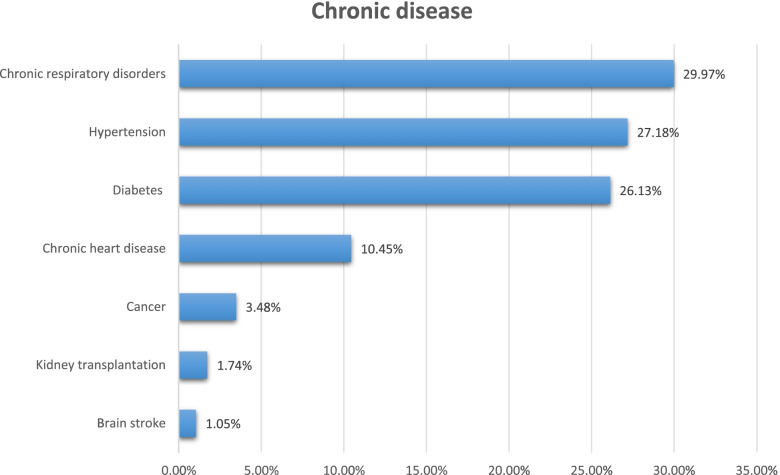
The distribution of the chronic disease among participants

**Fig. 2:**
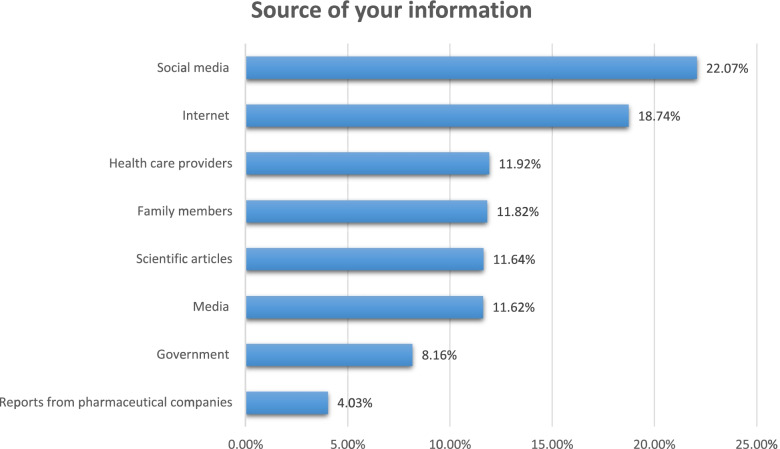
The most reliable sources of information about COVID-19 vaccines in Palestine

**Fig. 3:**
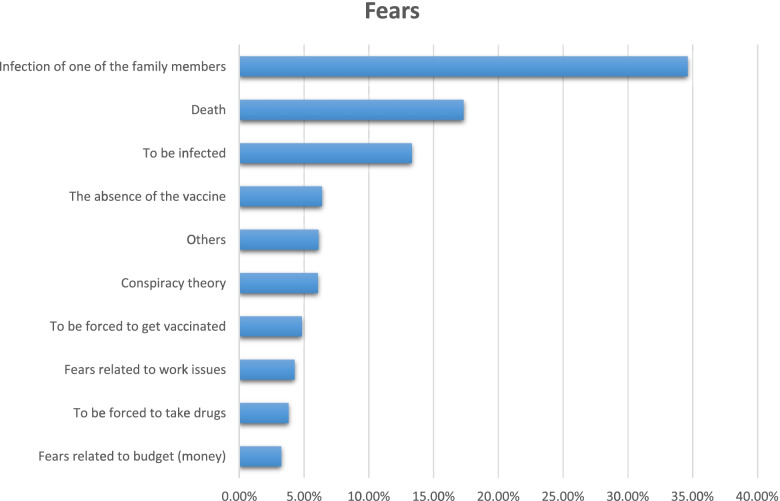
Palestinian fears during the COVID-19 pandemic

Among 237 participants who were reported with chronic diseases, more than 50% have chronic respiratory disorders and hypertension (29.97%) (27.18%), respectively (See Fig. 1). Regarding the source of information about COVID-19, social media, the internet, and health care providers were the primary source of information for the participants (22.07%), (18.74%), and (11.92%) respectively (See Fig. 2). Most of the participants' fears are related to death and family members’ infection (17.33%) and (34.62%), respectively (See Fig. 3).

### Knowledge of participants towards Covid-19 vaccinations

Frequencies of participants’ answers for each question regarding the knowledge about the COVID-19 vaccine is presented in Table 2. Gender, Governorate, Age group, Education level, Marital status, Previous COVID-19 Infection, and Vaccine administration were statistically significant (*P*-values<0.05). On the other hand, a history of chronic diseases showed no statistical difference in terms of the knowledge score (Table 3).

**Table 2:** Participants’ knowledge toward COVID-19

Statement	Yes N (%)	No N (%)	I don’t Know
There is an effective drug available to treat COVID-19.	1211 (20.3%)	2990 (50%)	1778 (29.7%)
Vaccines are safe.	2498 (41.8%)	1211 (20.3%)	2270 (38%)
There are ways to help slow the spread of the Coronavirus.	5100 (85.3%)	348 (5.8%)	531 (8.9%)
There is currently a vaccine to protect against Coronavirus infection.	4034 (67.5%)	921 (15.4%)	1024 (17.1%)
Regular flu vaccine will protect me from COVID-19.	284 (4.7%)	4034 (67.5%)	1661 (27.8%)
Antibiotics are an effective treatment for COVID-19	936 (16.7%)	3296 (55.1%)	1747 (29.2%)
Taking vitamin C or other vitamins will protect you from Coronavirus infection.	2802 (46.9%)	1867 (31.2%)	1310 (21.9%)
Wearing a mask helps reduce the spread of the Coronavirus.	5331 (89.2%)	411 (6.9%)	237 (4.0%)
Hand washing helps reduce the spread of the Coronavirus.	5481 (91.7%)	280 (4.7%)	218 (3.6%)
Cleaning surfaces helps reduce the spread of the Coronavirus.	5109 (85.4%)	513 (8.6%)	357 (6.0%)
Vaccines against pneumonia will protect you from the Coronavirus.	1016 (17%)	2059 (34.4%)	2904 (48.6%)
Regularly rinsing your nose with a saline solution will protect you from Coronavirus infection.	2021 (33.8%)	1602 (26.8%)	2357 (39.4%)
So far, no one in your country has died from the Coronavirus infection.	514 (8.6%)	5130 (85.8%)	336 (5.6%)
Eating garlic will protect you from the Coronavirus infection	1173 (19.6%)	2503 (41.9%)	2303 (38.5%)
COVID-19 health complications are more severe on people who already have a serious medical condition.	5016 (83.9%)	309 (5.2%)	654 (10.9%)
Other strains of Coronavirus can infect humans, including those that cause the common cold.	4230 (70.7%)	329 (5.5%)	1420 (23.7%)
The virus has been genetically modified as part of a biological weapons program	1547 (25.9%)	1037 (17.3%)	3395 (56.8%)
The virus is originally man-made and was spread intentionally	1665 (27.8%)	1272 (21.3%)	3042 (50.9%)

**Table 3:** Influence of Palestinian participants Socio-Demographic and professional characteristics on knowledge

Characteristic	Mean ± SD (out of 18)	*P*-value
Gender		
Male	10.23 ± 3.7	0.007
Female	10.55 ± 3.26	
The Governorate		
Gaza strip	10.98 ± 2.94	<0.001
West Bank	10.37 ± 3.45	
Age group		
18 – 19	10.21 ± 3.21	<0.001
20 – 24	10.88 ± 3.35	
25 – 29	10.16 ± 3.68	
> 29	9.67 ± 3.63	
Education level		
Non-educated	7.39 ± 3.27	<0.001
Primary school	8.84 ± 3.21	
Elementary school	7.21 ± 3.92	
Secondary school	9.3 ± 3.55	
University	10.58 ± 3.34	
Marital status		
Single	10.69 ± 3.32	<0.001
Married	9.72 ± 3.42	
Divorced	9.66 ± 4.73	
Widower	7.2 ± 4.15	
Chronic disease		
Yes	10.1 ± 3.67	0.076
No	10.48 ± 3.37	
Previous infected with the corona virus		
Yes	10.72 ± 3.27	<0.001
No	10.14 ± 3.51	
COVID-19 vaccinated		
Yes	11.2 ± 3.5	<0.001
No	10.34 ± 3.55	

### The attitude of participants towards Covid-19 vaccinations

Participants’ answers for the attitude towards the vaccine questions were reported in Table 4. Among participants, 55.1% agreed that they would administer the vaccine once available.

**Table 4:** Participants’ Attitude toward COVID-19

Statement	Agree N (%)	Disagree N (%)	Don’t know (Neutral) N (%)
It is important to get vaccinated to protect people from coronavirus infection.	4130 (69.1%)	561 (9.4%)	1288 (21.5%)
I have concerns about the unexpected effects of the vaccine.	3780 (63.2%)	1251 (20.9%)	948 (15.9%)
Pharmaceutical companies are developing safe and effective vaccines against coronavirus.	3304 (55.3%)	527 (8.8%)	2148 (35.9%)
I don't generally trust the benefits of the vaccine.	2232 (37.3%)	2593 (43.4%)	1154 (19.3%)
Coronavirus vaccines developed in Europe or the United States are much safer than those made in other countries of the world.	1547 (25.9%)	1623 (27.1%)	2809 (47%)
I have concerns regarding the commercial profitability of the pharmaceutical companies	2567 (42.9%)	1827 (30.6%)	1585 (26.5%)
Possible side effects are considered barriers for me to administer the COVID-19 vaccine.	1908 (31.9%)	2603 (43.5%)	1468 (24.6%)
Natural immunity is better than the immunity obtained by the COVID-19 vaccine.	3484 (58.3%)	1623 (27.1%)	872 (14.6%)
If you were offered the vaccine, would you accept it?	3295 (55.1%)	1668 (27.9%)	1016 (17%)

## Discussion

This is a cross-sectional study that was conducted among Palestinians in different areas, it has revealed that the general populations in the different areas of Palestine have good knowledge towards the preventive measures against the new coronavirus, which positively affects their attitudes to avoid spreading of infection. Regarding the attitudes and acceptance of COVID-19 vaccines among Palestinians; they have a low acceptance rate despite their knowledge that the vaccine can protect against COVID-19 infection.

Palestinians showed a good knowledge in the measures used in preventing the spread of the new coronavirus, 91.7%, 89.2%, and 85.4% agreed that hand washing, wearing masks, and cleaning surfaces reduce the spread of COVID-19 infection; respectively, participants from Gaza strip showed a better knowledge when being compared to participants from the West bank, and the majority of participants (85.3%) believed that there are ways that help to slow the spreading of the virus, which is consistent with a survey conducted in the United States and the United Kingdom found that respondents understood how COVID-19 is transmitted and disseminated [26], in addition, our results were consistent and similar to a previous study in a neighboring country, Jordan, which revealed that Jordanians had a positive attitude towards COVID-19; about 99.7% of the participants believed that hand washing is essential, 81.8% and 79.5% of participants agreed that smoking and antibiotics could not prevent infection [27].

Palestinians also believed that COVID-19 complications are more severe in patients with previous serious medical conditions (83.9%), about half of the participants believed that vitamin C or garlic could protect them from COVID-19 (46.9%) (19.6%), respectively, while more than half of participants believed that antibiotics won’t be helpful against the new coronavirus. More than half of Palestinians (67.54%) know that there is a vaccine against COVID-19, (41.8%) believe that the COVID-19 vaccine is safe, and (67.5%) and (34.4%) believe that the flu vaccine and the pneumococcal vaccine will not protect against COVID-19. When being compared with other populations, about a quarter of people in Bangladesh believe that the COVID-19 vaccine is safe [21]. In Jordan, 90% of participants clearly understood COVID-19 symptoms, and more than 80% were aware of the lack of vaccination [27]. Knowledge is vital in stopping spreading the coronavirus, a Chinese study has reported that knowledge has a direct impact on attitudes [28].

Palestinians have a good knowledge of preventive measures of COVID-19 which positively affects their attitudes to avoid the spread of infection. however, although (85.3%) of participants did not receive any vaccines, only (55.1%) reported that they would receive the vaccine if they had a chance to. The low percent of Palestinians, as reported in this study, who administered the vaccine can be explained mainly by the low percentage of vaccines acceptance as revealed by our study, also other reasons may contribute like the unavailability of vaccines or weaknesses of healthcare authorities to promote the vaccines availability, although (69.1%) believe that getting the vaccine would protect other people from having COVID-19.

Just like the previous pandemics humanity has faced, the COVID-19 pandemic is accompanied by feelings of fear, anxiety [29, 30]. Our findings agreed with those of Mertens et al., who reported that higher levels of fear during the COVID-19 pandemic were linked to family members getting infected or dead [31]. The self-worry is focused on preventing the disease from spreading to family members, particularly the elderly who have been recognized as vulnerable to bad COVID-19 prognosis [32]. Further widely, surveys that investigated the association between vaccination willingness and fear of the disease, found that individuals who are fearful of the disease are more likely to be vaccinated [33–37].

Studies have found that a people's intention to accept the vaccine is influenced by a variety of variables, including the risk of viral infection, the severity of the viral complications, the vaccine's safety, efficacy, and adverse reactions, misinformation, misconceptions regarding the vaccine, and lack of understanding the nature of diseases which are vaccine-preventable [38, 39].

There are conflicting reports of gender effects in the literature, some reported that males were more acceptable to receiving the vaccine in comparison with females [40, 41], in contrast, other studies indicated that females were more acceptable to receive the vaccine [42, 43]. In this study, the knowledge score for females’ participants was higher than the knowledge score among males’ participants. When being compared to other countries, Palestinians have a lower acceptance rate towards COVID-19 vaccine when being compared to other countries, for instance, Saudi Arabia, recorded a higher acceptance rate of (64.7%) [43], while in China acceptance rate was (88.6%) [44].

We found that younger participants had a better knowledge than older participants, contrary to previous research that found older age groups to be more accepting [41, 43, 44]. Differences in age distribution among some countries can explain those results, while Palestine is considered a country with a predominantly young population. In addition, participants with higher education (bachelor degree or above) are found to have higher knowledge score when compared with participants with lower education., which is consistent with a study conducted among six countries which indicates a relationship between good knowledge and level of education [45].

We stated that (55.3%) of the participants agreed that pharmaceutical companies would develop safe and effective COVID-19 vaccines, however, (63.2%) and (42.9%) reported some concerns about the vaccine side effects and the commercial profitability of the pharmaceutical companies; respectively. This discrepancy could be attributed to a global phenomenon that has contributed to such a low level of trust as various anti-vaccination campaigns fueled by modern technologies of communication; social media with fake, incorrect, and often inaccurate Arabic content and translations feed some people's conspiracy theories. Some factors associated with the country and region may also have a role, for example, Palestinian people can have lower confidence in the Palestinian authorities, some reports indicated that there is a positive correlation between lower acceptance levels of COVID-19 vaccines and lower confidence in the government's ability to handle the pandemic [44, 46].

Understanding the sources of information about COVID-19 vaccines that people trust the most is critical to future national vaccination programs [47, 48]. When Palestinians were asked which sources of information regarding COVID-19 vaccines they used the most, social media came out on top followed by the internet, while the government and pharmaceutical companies were the least trusted information sources, which is consistent with Malik Sallam et. al. findings that social media is the main source of information among participants who have higher Vaccine Conspiracy Belief Scale (VCBS) scores [49].

Good knowledge about COVID-19 vaccines is vital to increase the rate of acceptance to administer the vaccine, therefore, it is important to provide a reliable and scientific source of information which is evidence-based to be disseminated in the press and media to reach the public. Our study has shown that the largest percentage of knowledge among Palestinians is gained from social media and internet, therefore, attention must be paid to publishing scientific evidence-based content for people on internet and different social media platforms, also, Palestinian authorities should fight rumor mongers and limit their impact on society, as well as educating the media cadre about vaccines in order to involve them in urging people to take the vaccine and reassured public about the vaccine’s effectiveness and side effects. Our study showed that there are many non-vaccinated populations who have intentions to take the vaccine if it was available, we believe that the Palestinian Ministry of Health should target this large segment, and this could be done through the organization of vaccination campaigns.

Our study will help health care providers and policymakers in Palestine to understand public knowledge, attitude and acceptance toward COVID-19 vaccines and to take appropriate procedures and interventions aimed to increase the rate of vaccine acceptance and improving the health sector. It is also useful to carry out longitudinal studies aimed to study the behavior of Palestinians more accurately so that we can take the appropriate and rational action.

### Strengths

- We collected a large sample size from Palestinian individuals from various academic levels.
- The current findings describe the public reactions of Palestinians towards the COVID-19 vaccines.
- The results provide a good beginning point for identifying demographics and factors involving in affecting health behaviors.
- The pattern of results could be generalized to a larger population.
- Self-report measures were primarily employed to optimize convenience sampling to avoid bias effects.

### Limitations

- Online questionnaire may missed elderly people or people who have no internet access.
- Our study is an online cross-sectional, so it is susceptible to participation bias.

## Conclusion

Palestinian populations could have good knowledge and attitudes towards COVID-19, although they have low acceptance rate towards COVID-19 vaccines compared to other countries. Public health authorities should promote more research into the core causes of the differences in the acceptance rate towards COVID-19 vaccines worldwide. National health authorities should re-establish trusted sources of information as campaigns to provide more information about the safety, efficacy, and technology used to produce vaccinations.

## Data Availability

The datasets used and/or analyzed during the current study are available from the corresponding author on reasonable request.
